# Estimation of serum lipids in patients with Oral Submucous Fibrosis in India

**DOI:** 10.4317/jced.51327

**Published:** 2014-07-01

**Authors:** Kratika Ajai, Sunil R. Panat, Ashish Aggarwal, Nupur Agarwal, Nitin Upadhyay, Anuja Joshi

**Affiliations:** 1 Post Graduate Student. Department of Oral Medicine and Radiology, Institute of Dental Sciences, Bareilly, UP, India; 2 Professor and Head. Department of Oral Medicine and Radiology, Institute of Dental Sciences, Bareilly, UP, India; 3 Senior Lecturer. Department of Oral Medicine and Radiology, Institute of Dental Sciences, Bareilly, UP, India

## Abstract

Objectives: Oral submucous fibrosis (OSMF) is the most prevalent precancerous condition in India. Low levels of lipids serves as a marker and prognostic indicator in the early detection of oral precancerous and cancerous states. In spite of the high prevalence and its potential to undergo malignant transformation, this condition has not widely been investigated with respect to the serum lipid levels. In the present study, an attempt was made to analyze the complete serum lipid profile, total cholesterol (TC), triglycerides (TG), high density lipoprotein (HDL) cholesterol, low density lipoprotein (LDL) cholesterol and very low density lipoprotein (VLDL) cholesterol in OSMF and controls. Material and Methods: The study was conducted in 45 clinically and histopathologically diagnosed cases of OSMF and 45 age and sex matched controls. The complete lipid profile including TC, TG, HDL cholesterol, LDL cholesterol and VLDL cholesterol was analyzed. Results: The serum lipid levels were significantly lower in the patients with OSMF than in the controls. When the values were compared between different disease stages, the maximum reduction of lipids was evident for stage 3 OSMF. From the present results, it is evident that the level of serum lipids decreases with progression of the disease. Conclusions: From these findings, it appears that the decrease in the lipid levels may be considered as a useful marker in the early diagnosis of oral premalignant condition like OSMF.

** Key words:**Oral submucous fibrosis, lipids, premalignant condition.

## Introduction

Oral submucous fibrosis [OSMF] is a chronic debilitating disease and a premalignant condition of the oral cavity (1). Oral cancer is the leading cause of morbidity and mortality in India and is most commonly preceded by clinically definable premalignant lesions and conditions (2). OSMF is regarded as a precancerous condition and shows a significant tendency to develop cancer (3).

Over the years, the incidence of OSMF has increased in various parts of the Indian subcontinent (4). Murti *et al.* (5) demonstrated a malignant transformation rate of 7.6% over a 17-year observation period. Therefore, it is also equally important to detect and control the premalignant lesions and conditions (3). Early detection of these conditions can dramatically improve the treatment outcome and prognosis in such patients (2).

In recent years, emphasis has been placed on detecting molecular markers from body fluid, such as saliva, urine and others, for detecting cancer, predicting prognosis, and monitoring disease progression. The idea of screening and following patients with malignancy by blood-based tests is appealing from several points of view including its ease, economic advantage, non-invasiveness, and possibility of repeated sampling (6).

Usefulness of variations in tissue ⁄ blood cholesterol levels in diagnosis and treatment of various diseases has been studied by several workers. Although, its prime role in pathogenesis of coronary heart diseases has been consistently found, researchers have reported an association of plasma ⁄ serum lipids and lipoproteins with different cancers (7-10). An alteration in the circulatory cholesterol levels has been found to be associated in the etiology of breast cancer and colorectal cancer (11-13). Lipids may not play a role in carcinogenesis but a lower level of lipids may indicate rapidly dividing cells in malignancy. This can serve as a marker in early neoplastic changes, in follow up cases and as a prognostic indicator of disease. The mechanism for the association of cancer with cholesterol remains controversial. The exact mechanism of its role in carcinogenesis is not clearly defined. Cell membrane is essential for cell survival as well as biological functions (14). It is believed that tobacco carcinogens induce generation of free radicals and reactive oxygen species, which are responsible for high rate of oxidation ⁄ peroxidation of polyunsaturated fatty acids. This peroxidation further releases peroxide radicals. This affects essential constituents of cell membrane and might be involved in carcinogenesis ⁄ tumorigenesis (15). Lipids play a key role in maintenance of cell integrity. Because of lipid peroxidation, there is a greater utilization of lipids including TC, lipoproteins, and triglycerides for new membrane biogenesis. Cells fulfill these requirements from circulation either by synthesis through the metabolism or from degradation of major lipoprotein fraction such as VLDL, LDL, or HDL (16).

Rose *et al.* (17) reported an inverse association between blood cholesterol level and the risk of cancer and provided a base for further epidemiological research. Since then, conflicting hypotheses have been put forward by many workers. Several authors propose that hypocholesterolemia is a predisposing factor for cancer development (18). Only a few reports are available on plasma/serum lipid profile in head and neck lesions (16). However, these previous studies were limited by the fact that they evaluated lipid profile only in cancer patients, whereas the present study evaluated lipid profile in the oral premalignant condition like OSMF.

In spite of the high prevalence and its potential to undergo malignant transformation, OSMF has not widely been investigated with respect to the serum lipid levels. Moreover, to our knowledge, literature pertaining to serum lipid levels in relation to OSMF and its clinical grading is rare. In the present study, an attempt was made to analyze the complete serum lipid profile in OSMF patients. Further, in this study the comparison in the serum lipid profile with grading in OSMF was done.

## Material and Methods

The study was conducted in 45 clinically and histopathologically diagnosed cases of OSMF who attended the Department Of Oral Medicine and Radiology, Institute Of Dental Sciences, Bareilly, Uttar Pradesh-India. All the patients underwent recording of detailed history, and clinical diagnosis was made on the presence of characteristic features like vesicles, ulceration, mucosal blanching, burning, stiffness of oral mucosa, presence of characteristic fibrous bands and progressive inability to open the mouth. Also, histopathological examination was carried out in all the cases following incisional biopsy from the most affected area of the buccal mucosa. OSMF was divided clinically and functionally into three stages according to the criteria reported by Haider *et al.* (19), based on the presence of fibrous bands at various anatomical sites, and functional staging was based on the degree of mouth opening.

Clinical Staging:

• Stage 1: Faucial bands only

• Stage 2: Faucial and buccal bands

• Stage 3: Faucial, buccal and labial bands

Functional Staging:

• Stage 1: Mouth opening >20 mm

• Stage 2: Mouth opening 11-19 mm

• Stage 3: Mouth opening <10 mm

45 healthy individuals, matched for age and sex, who consecutively presented at the same department for various other complaints, and had no adverse habits or oral lesions, or any other major illness in recent past, were included as controls. Informed consent was obtained from patients in both the case and control groups. The study was approved by the institutional ethical committee.

Exclusion criteria were patients with systemic diseases/conditions that may be associated with alterations in the serum level of lipid profile like obese subjects weighing more than 20% above their ideal weight, known cases of uncontrolled diabetes mellitus, thyroid disorder, cardiac patients, liver dysfunction, malabsorption syndrome or those refusing for biopsy procedure and patients who had undergone or was on treatment for OSMF.

The subjects of the study were as follows:

• Group I [Clinical and functional stage 1 OSMF]: 15 patients

• Group II [Clinical and functional stage 2 OSMF]: 15 patients 

• Group III [Clinical and functional stage 3 OSMF]: 15 patients

• Group IV [Controls]: 45 patients

All those patients who fulfill the above mentioned criteria were included in the study.

- Estimation of lipid profile

First, the details of each patient, including medical history, were taken. After a clinical diagnosis of OSMF had been made, the purpose and procedure was explained to each patient, who then provided informed consent to participate. The patient was then asked to attend the next morning with a minimum of 12 hour of fasting for blood examination for complete lipid profile. Upon returning, the patient was asked to sit comfortably in a dental chair in a reclining position. A tourniquet was then applied above the right cubital fossa, and 3 ml of fas-ting blood was collected from the most prominent vein under aseptic conditions and was stored in test tubes. After the blood had coagulated, the test tube containing the blood was subjected to centrifugation for about 4-5 min at 2500 rpm. The test tube was then removed from the centrifuge, and the serum layer was pipetted into a vial, which was then stored in a refrigerator under protection from light. The serum lipid profile was analyzed using the kit Erba Mannheim [Transasia bio-medicals limited, Solan, India] as per instructions provided by the manufacture, using semi-auto analyser. Complete lipid profile was checked in each case. TC, TG, HDL choles-terol, LDL cholesterol and VLDL cholesterol were monitored using Modified Roeschlau’s method for cholesterol, method of Wako and the modifications by McGowan *et al* and Fossati *et al* for triglycerides and Phosphotungstic Acid method for HDL.

VLDL and LDL levels were calculated using Friedwald’s formula:

• VLDL= Triglycerides/5

• LDL= Total Cholesterol – [VLDL+HDL]

- Statistical analysis

All the variables from the study were statistically analyzed using the Statistical Package for the Social Sciences program [SPSS version 13.0]. Analysis of variance [ANOVA] was used to assess the statistical significance of differences between the stages of OSMF and the control group. Student’s t-test was used to compare serum lipid profile in all the three case groups with those in the control group.

## Results

The present study comprises of 90 subjects, of whom 64 were males and 26 were females. Group IV comprised of 45 age and sex matched healthy individuals without any contributory habits or premalignant lesions/conditions. Group I, II, III comprised of 11, 10, 12 males and 4, 5, 3 females, respectively. The mean age of the patients in group I, II, III and IV was 25.13 years, 27.60 years, 34.53 years and 29.09 years respectively with most of the patients in the second and third decades of life. All of the patients had been taking areca nut in one form or another. The mean serum TC, serum HDL cholesterol, serum LDL, serum VLDL and serum TG levels in oral submucous fibrosis group were 151.07 mg/dL, 37.80 mg/dL, 95.15 mg/dL, 18.11 mg/dL and 94.18 mg/dL respectively. However, in the control group the corresponding values were 191.42 mg/dL, 51.36 mg/dL, 113.64 mg/dL, 26.43 mg/dL and 132.16 mg/dL respectively. ( Table 1) A statistically significant reduction [P<0.001] was noted between the control group and OSMF cases.

**Table 1:**
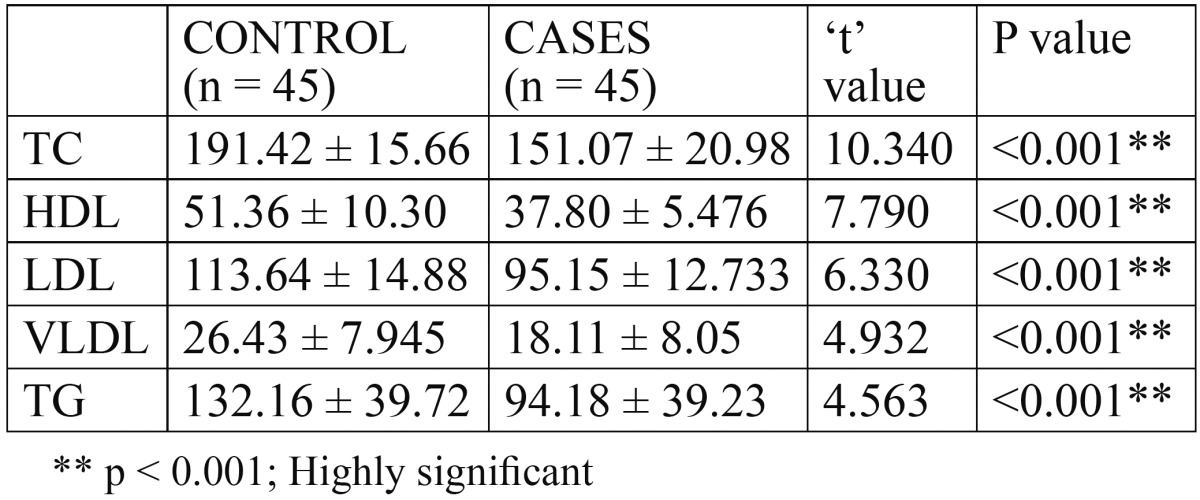
Comparison of mean serum lipid profile among control group and OSMF cases.

Similarly, in different stages of OSMF i.e stage 1, stage 2 and stage 3, these values were 174.20 mg/dL, 152.27 mg/dL and 126.73 mg/dL for TC; 42.47 mg/dL, 38.20 mg/dL and 32.73 mg/dL for HDL; 106.04 mg/dL, 97.27 mg/dL and 82.15 mg/dL for LDL; 25.69 mg/dL, 16.80 mg/dL and 11.85 mg/dL for VLDL; and 128.47 mg/dL, 92.80 mg/dL and 61.27 mg/dL for TC, respectively. These values were found to decrease progressively as one moved from control group to that of OSMF cases. It also shows that as the disease progresses i.e from OSMF stage 1 to stage 3, there is a gradual fall in lipid profile levels ( Table 2).

**Table 2:**
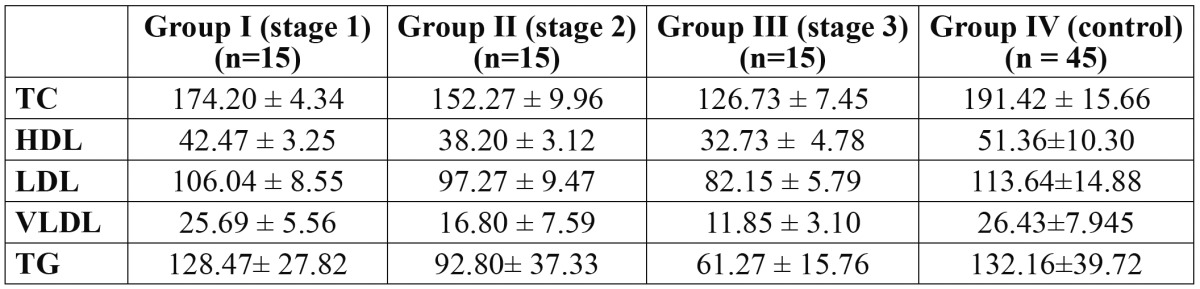
Mean serum lipid profile among control group and different stages of OSMF.

In inter-group and intra-group comparisons of serum lipid, for all variables p-value using ANOVA showed that four groups differ significantly that is non-homogeneity for the means of four groups ( Table 3).

**Table 3:**
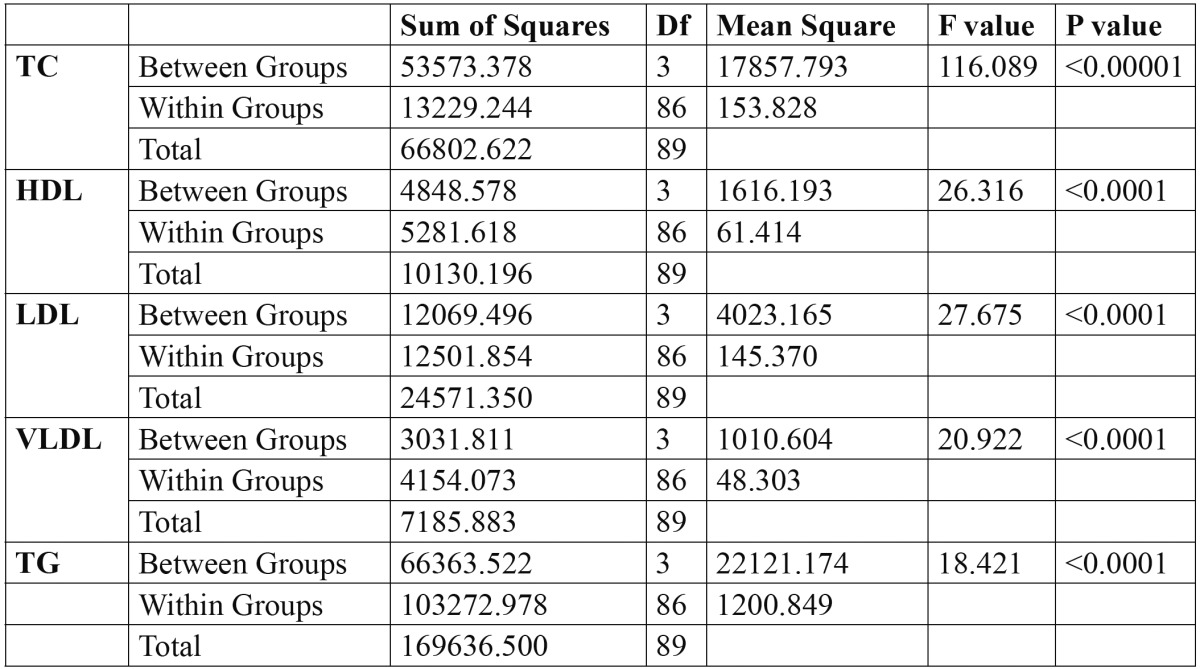
ANOVA test for serum lipid profile among various groups.

For pair-wise comparison a Post-hoc Tukey test was conducted. It showed that for TC all pairwise differences were statistically significant. For HDL all pairwise difference was statistically significant except for Group I & II and Group II & III. For LDL all pairwise differences were statistically significant except for Group I & IV and Group I & II. For both VLDL and TG all pairwise differences were statistically significant except for Group I & IV and Group II & III ( Table 4).

**Table 4:**
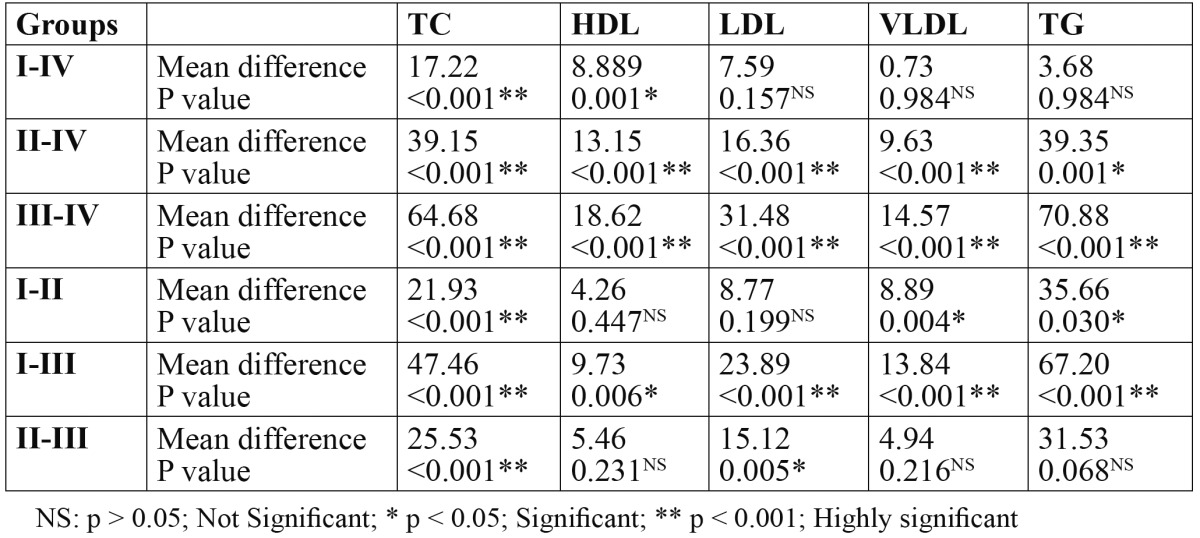
Multiple comparison of serum lipid profile among various groups using Post-Hoc Tukey test.

## Discussion

Oral submucous fibrosis is a chronic, insidious, disabling precancerous condition of the oral cavity (20). It has always been a challenging disease with high prevalence in India (21). In spite of the high prevalence of premalignant condition like OSMF in India and their potential to undergo malignant transformation, this condition has not widely been investigated with respect to the serum lipid levels. Early detection is also called as secondary prevention. It is therefore important to identify new diagnostics and predictive approaches.

In this study, an attempt was made to analyze serum lipid levels in patients with OSMF in comparison with normal healthy individuals, thereby providing a marker and prognostic indicator in the early detection of oral cancer. An attempt was also made to compare these levels among different stages of the disease.

The mean age of our study patients was 29.09 years. Most of our patients were in the second and third decades of life. Among our 45 OSMF patients, 32 were males and 13 were females. All the patients in our study had been chewing areca nut in some form. Areca nut chewing has been identified as the most important etiological factor of OSMF. A statistically significant reduction [P<0.001] was noted in serum TC, serum TG, serum HDL, serum LDL and serum VLDL between the control group and OSMF cases. Similarly, in different stages of OSMF i.e stage 1, stage 2 and stage 3, these values were found to decrease progressively as one moved from control group to that of OSMF cases. It also shows that as the disease progresses i.e from OSMF stage 1 to stage 3, there is a gradual fall in lipid profile levels. The reduced levels of serum lipid profile in OSMF patients may be a conse-quence of disease, probably mediated by the greater utilization of lipids for new membrane biogenesis.

Tilakaratne et al. (22) reported that areca nut is the main etiological factor for OSMF. Excessive use of areca nut may cause fibrosis due to increased synthesis of collagen and induce the production of free radicals and reactive oxygen species, which are responsible for high rate of oxidation/ peroxidation of polyunsaturated fatty acids which affect essential constituents of cell membrane and might be involved in tumorigenesis (21). Because of the lipid peroxidation, there is a greater utilization of lipids including lipoproteins, total cholesterol and triglycerides for new membrane formation. Cells fulfill these requirements either from circulation or from degradation of major lipoprotein fractions like VLDL, LDL or HDL (16). Lower levels of lipids thus result from increased utilization of lipids by the tumor cells for synthesis of cell membrane.

The results of the present study clearly shows that the serum TC, HDL, LDL, VLDL and TG were significantly reduced in OSMF patients group when compared with the control group. Patel *et al.* (16) showed the similar observations in patients with oral squamous cell carcinoma. They found a significant decrease in plasma TC, HDL, VLDL and TG but LDL cholesterol levels did not reveal any significant difference, whereas in our study significant decrease in LDL levels in OSMF was seen as compared to controls. Similarly, Lohe *et al.* (3) reported a significant decrease in TC, and HDL in oral precancerous conditions, however, LDL, VLDL, and triglyceride did not reveal any significant difference.

## Conclusions

The results of the present study show the evidence of an inverse relationship between serum lipid profile and OSMF. From these findings, it appears that the decrease in the lipid levels may be considered as a useful marker in the early diagnosis of oral premalignant condition like OSMF. Lipid profile can be added on to other tests as an additional indicator and can serve as another evaluating parameter to denote initial changes occurring during carcinogenesis. However, this will also have to be confirmed by using a larger sample size.
